# Plant Natural Products for Human Health

**DOI:** 10.3390/ijms20040830

**Published:** 2019-02-15

**Authors:** Chun-Tao Che, Hongjie Zhang

**Affiliations:** 1 Department of Medicinal Chemistry and Pharmacognosy, and the World Health Organization Collaborating Center for Traditional Medicine, College of Pharmacy, University of Illinois at Chicago, Chicago, IL 60612, USA; 2 School of Chinese Medicine, Hong Kong Baptist University, Kowloon, Hong Kong, China; zhanghj@hkbu.edu.hk

**Keywords:** plant natural product, drug discovery, human health

## Abstract

The aim of this Special Issue on “Plant Natural Products for Human Health” is to compile a series of scientific reports to demonstrate the medicinal potential of plant natural products, such as in vitro and in vivo activities, clinical effects, mechanisms of action, structure-activity relationships, and pharmacokinetic properties. With the global trend growing in popularity for botanical dietary supplements and phytopharmaceuticals, it is hoped that this Special Issue would serve as a timely reference for researchers and scholars who are interested in the discovery of potentially useful molecules from plant sources for health-related applications.

Plants have served mankind as an important source of foods and medicines. While we all consume plants and their products for nutritional support, a majority of the world population also rely on botanical remedies to meet their health needs, either as their own “traditional medicine” or as “complementary and alternative medicine” [1,2]. Today we are witnessing a global resurgence in interest and use of plant-based therapies and botanical healthcare products. For example, herbal supplement sales in the U.S. increased 8.5% (compared to the previous year), reaching an estimated total of 8 billion USD in 2017 [3]. The increased interest in herbal medicine and products by the general public has stimulated a greater scientific awareness in exploring and understanding the pharmacologically active constituents of medicinal plants.

From the pharmaceutical point of view, many compounds obtained from plant sources have long been known to possess bio/pharmacological activities, and historically plants have yielded many important drugs for human use, from morphine discovered in the early nineteenth century to the more recent paclitaxel and artemisinin. Although most mega pharmaceutical companies are not focusing on developing natural drugs at this time, natural products remain an important and viable source of lead compounds in many drug discovery programs. After all, development of natural products for the prevention and treatment of diseases continue to attract worldwide attention.

This Special Issue on “Plant Natural Products for Human Health” is intended to be a compilation of scientific reports to cover different aspects of biologically active plant natural products, such as chemical characterization, in vitro and in vivo activities, clinical effects, mechanism of action, structure-activity relationship, and pharmacokinetic/pharmacodynamic properties. With the global trend growing in popularity for botanical dietary supplements and plant-based drugs, it is our hope that this Special Issue would serve as a reference for researchers and scholars who are interested in the discovery of potentially useful molecules from plant sources for health-related applications.

We were overwhelmed to receive many high-quality manuscripts from all over the world. A total of 26 articles have been published in this Special Issue in 2018, covering a wide range of disease targets such as diabetes, inflammation, cancer, neurological disease, cardiovascular disease, liver damage, bacterial and fungus infection and malarial. They provide important insights into the current state of research on drug discovery and new techniques in the following areas of plant natural products.

In the diabetes area, Kang et al. reported that the Amadori rearrangement compounds obtained from heat-processed onion extract were able to suppress carbohydrate absorption through inhibition of intestinal sucrose, thereby reducing the postprandial increase of blood glucose [4]. On the other hand, Nagappan et al. reported the in vitro protective activity of gomisin N (obtained from *Schisandra chinensis*) against cannabinoid type-1 receptor-induced impairment of insulin signaling, as well as the in vivo effect of the compound on gluconeogenesis in high-fat-diet-induced-obese mice [5].

The neurological effects of natural products are demonstrated in three reports. Thus, Li et al. described the neuroprotective activity of phenylethanoid glycosides such as salidroside, acteoside, isoacteoside, and echinacoside, which were found to interfere with the Nrf2 binding site in Keap1 protein in the adrenal phaeochromocytoma PC12 cells [6]. Gugliandolo et al. reported the anti-inflammatory and anti-oxidant effects of cannabigerol (obtained from *Cannabis sativa*) on NSC-34 motor neurons, as demonstrated by a reduction in the IL-1β, TNF-α, IFN-γ and PPARγ protein levels [7]. Furthermore, Tang et al. demonstrated that catechin and procyanidin A2 (obtained from lychee seed) were able to suppress neuroinflammation in amyloid-β-treated microglial BV-2 cells [8].

Asperuloside and asperulosidic acid were reported by He et al. to possess anti-inflammatory activity in lipopolysaccharide-treated RAW 264.7 macrophages through suppression of the NF-κB and MAPK signaling pathways [9]. A glucan fraction prepared from the stalk of *Pleurotus eryngii* mushroom was also shown by Vetvicka et al. to suppress inflammation in a dextran sulfate sodium-induced mouse model of inflammatory bowel disease. The glucan fraction displayed downregulation effects on IFN-γ and MIP-2 levels [10].

In cancer studies, the potential of hinokitiol in lung cancer chemoprevention was described by Jayakumar et al. The compound was shown to inhibit the migration of lung adenocarcinoma A549 cells through several pathways, such as activation of capases-3 and -9, induction of p53/Bax and the antioxidant enzymes CAT and SOD, as well as reduction of MMP-2 and -9 activities [11]. In another study, Zheng et al. demonstrated that the flavonoids isolated from *Glycyrrhiza uralensis* (Chinese liquorice) could induce differentiation of melanoma B16-F10 cells or promote apoptosis [12].

Four papers have focused on cardioprotection. In a study by Czompa et al., the effects of raw and aged black garlic were compared using a rat model of post-ischemic cardiac recovery. Both types of garlic were found to display cardioprotective activity as demonstrated by an enhancement of post-ischemic cardiac function and a reduction in cardiac infarct size [13]. In another study, Hsia et al. reported that morin hydrate could inhibit platelet activation through an inhibition of the PLCγ2-PCK cascade and subsequent suppression of Akt and MAPK activation [14]. On the other hand, Guo et al. reported dihyromyricetin being able to ameliorate myocardial hypertrophy in a transverse aortic constriction mouse model, and the authors suggested that the activity of the compound was related to the suppression of oxidative stress and an upregulation of the SIRT3 pathway [15]. Moreover, Hung et al. reported that the polar extract and chemical ingredients (such as astragaloside IV) of *Astragalus membranaceus* root, which is often used as a tonic herbal drug, exhibited a protective effect on cardiomyocytes exposed to oxidative stress through an increase in the respiratory capacity and mitochondrial ATP production [16].

Liver protection formed the theme of three papers. Using a hepatic steatosis model of HepG2 cells treated with free fatty acids, Guo et al. described the preventive activity of pinocembrin and its glucosides against hepatic steatosis, possibly through the regulation of the SIRT1/AMPK pathway [15]. In another study, fucoidan, a sulphated polysaccharides found in seaweeds, was reported by Wang et al. to be able to protect against hepatotoxicity induced by acetaminophen in a mouse model, with a plausible mechanism related to Nrf2-mediated oxidative stress [17]. Furthermore, the pharmacokinetics of the hepatoprotective triterpenic acids obtained from *Ziziphus jujube* fruits was reported by Li et al. using an UHPLC-MS method to analyze the plasma samples in normal and CCl_4_-treated rats [18].

In a screen of phytochemicals isolated from Himalayan medicinal plants, Wangchuk et al. reported scoulerine and bergapten as immunomodulators [19]. A series of cinnamamide derivatives were reported by Pospisilova et al. to possess significant antibacterial, antitubercular, and antifungal activities [20].

The herb-drug interaction between *Panax notoginseng* saponins and aspirin was investigated by Subramanya et al. The saponins were found to inhibit aspirin hydrolysis in HepaRG cells, the effect being ascribed to a suppression of the carboxylesterase-2 enzyme [21].

There are a total of seven review articles included in this Special Issue. They cover various biomedical areas including antimalarial (Pan et al., [22]), anti-arthritic (Dudics et al., [23]), hair growth stimulating (Choi, [24]), and anti-hepatotoxic (Subramanya et al., [21]) properties. Two other reviews have focused on the medicinal plants of *Copaifera* (da Trindade et al., [25]) and *Citrus* species (Dosoky and Setzer, [26]), respectively. Lastly, a review by Thomford et al. discussed the potential of applying innovative technologies such as automation technology, analytical and computational techniques to the next generation of plant-based drug discovery [27].

In conclusion, it is clear from these studies that many plant natural products display interesting bio/pharmacological activities. To better understand their medicinal properties and to establish stronger evidence of potentials for further development, preclinical and clinical investigations regarding their mechanisms of action, safety and efficacy are warranted.
